# A photograph of the researcher on the invitation letter did not affect the participation rate of a postal survey: a randomized study within a trial (SWAT)

**DOI:** 10.1186/s12874-022-01717-3

**Published:** 2022-09-24

**Authors:** Barbara Prediger, Nadja Könsgen, Ana-Mihaela Bora, Anna Schlimbach, Dawid Pieper

**Affiliations:** 1 Institute for Research in Operative Medicine, Witten/Herdecke University, Cologne, Germany; 2 Faculty of Health Sciences Brandenburg, Brandenburg Medical School Theodor Fontane, Institute for Health Services and Health System Research, Rüdersdorf, Germany; 3 Center for Health Services Research, Brandenburg Medical School Theodor Fontane, Neuruppin, Germany

**Keywords:** Recruitment, Participation rate, Enhancement strategies, Study within a trial, Personalization

## Abstract

**Objective:**

Participant recruitment is one of the main challenges in research. It is suggested that including researcher photographs might increase participation rates, but empirical evidence is lacking. This study within a trial (SWAT) aims to assess whether invitation letters including researcher photographs increase the participation rate in the context of a survey on medical second opinions.

**Methods:**

Through 25 local register offices in Berlin and Brandenburg (Germany), we identified a random sample of 9990 persons. We randomly assigned our sample to the intervention group (IG) receiving an invitation letter with researcher photographs and control group (CG) receiving an invitation letter without photographs in a 1:1 ratio. Our primary outcome was the participation rate. Furthermore, we compared participants to non-participants’ characteristics.

**Results:**

Of 9990 invitations, 9797 could be delivered (IG: 4890, CG: 4907). Of these, 1349 (13.8%) participated. There were 682/4890 (13.9%) participants in the IG and 662/4907 (13.5%) in the CG with an odds ratio of 1.030 (95% confidence interval: 0.918–1.156). Additional analyses on non-participant characteristics did not show any differences.

**Conclusion:**

We could not find any difference in the participation rates. Our study does not confirm the results of previous studies. The length of our questionnaire may have affected our results.

**Trial registration:**

Queens University Belfast – SWAT Store, SWAT 104.

**Supplementary Information:**

The online version contains supplementary material available at 10.1186/s12874-022-01717-3.

## Background

Recruitment is one of the main issues in any research that depends on participant involvement. Only 55% of trials recruited their estimated sample size according to two funding agencies from the United Kingdom [1]. Unsurprisingly, “Research into methods to boost recruitment in trials” was rated to be the top priority research in a survey on methodological research prioritization among directors of clinical trial units in 2012 [2]. The problem of recruitment does also arise in survey research. There are numerous ways of enhancing the participation rate in surveys [3]. Effectiveness of these enhancement strategies does not only rely on the type of survey (postal, by phone or electronic) but also on the sender or the receiver of the survey. In addition to monetary and non-monetary incentives in the form of direct compensation or the chance to win a lottery prize, various strategies might have a more subtle effect. The focus can be on the relationship with the sender, the length, appearance and style of the questionnaire, but also on low-threshold responses (e.g., stamped envelopes) [4–9]. Enhancement strategies that were found to be effective often result in high costs. It is most effective to pay prepaid cash incentives to the recipients of the survey [10]. The use of stamped envelopes for reply instead of business-reply envelopes is more effective but leads to higher costs, too [11, 12]. In a university setting with low financial resources, appearance of the questionnaire, envelope or invitation letter and building up a relationship between sender and receiver through personalization might be a solution. Personalization can be a personalized salutation or a handwritten signature, but also a photograph or personal note of the researcher [13]. Nevertheless the design should prevent that those who choose to participate in a survey may have systematic different characteristics (including attitudes toward participating in a survey) than those who refuse to participate to avoid selection bias [14].

A thorough Cochrane review found that there was convincing evidence only for the length of a questionnaire (short questionnaires increase participation rates), but not for the effects of other strategies [12]. Hence, we chose a personalization strategy, which does not cost additional money and other resources, to analyze the effect on the participation rate in a large sample. Our sample was drawn from the general population. The aim of this study within a trial (SWAT) was to compare the participation rate resulting from different ways to contact the participants (photograph vs. no photograph) in a survey regarding attitudes towards medical second opinions and whether there are sociodemographic subgroups for which the intervention has a larger effect.

## Methods

### Registration

This SWAT was registered in Queens University Belfast – SWAT Store, as SWAT 104 (04/10/2019).

### Setting

**Fig. 1:**
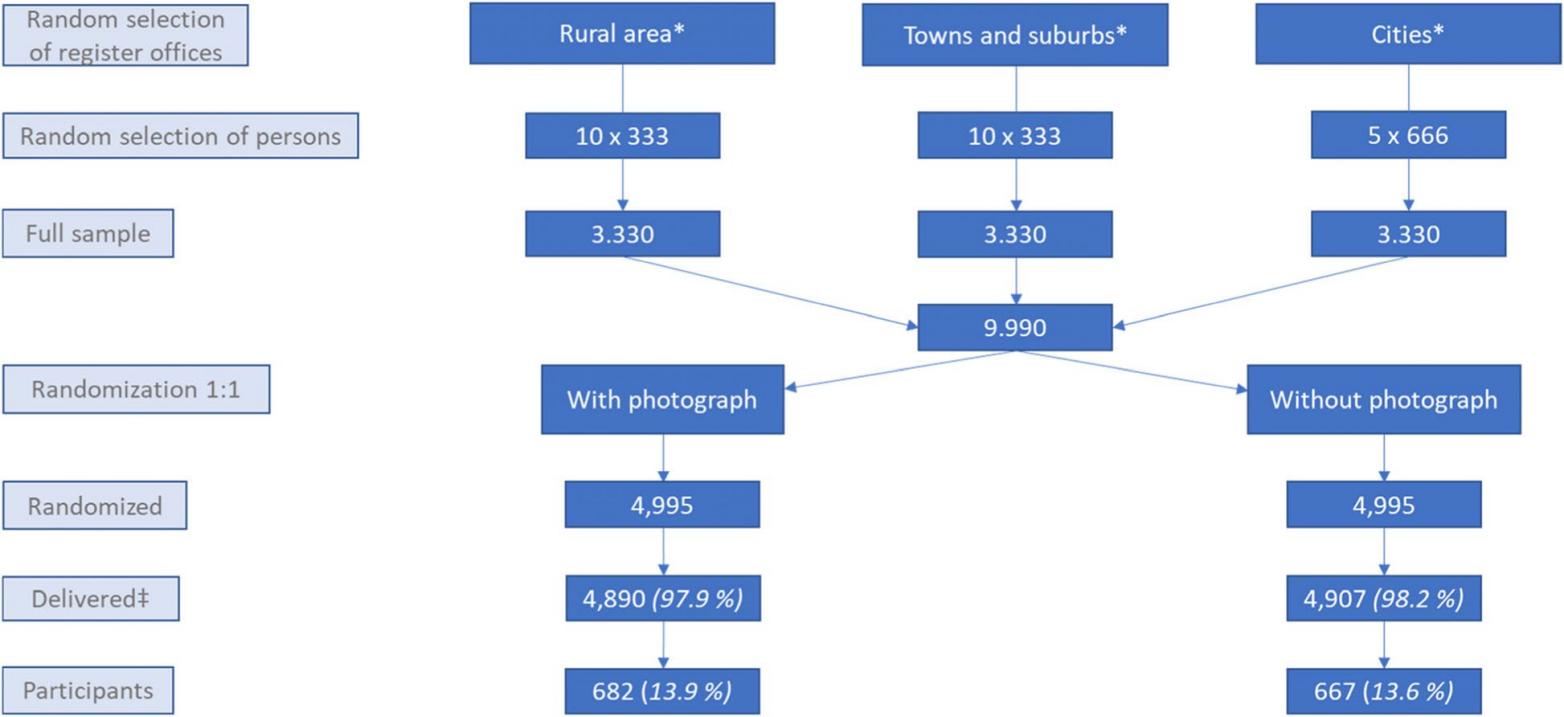
Participant flow chart. *Defined by Eurostat. ‡It was not possible to determine if a questionnaire was successfully delivered to the recipient

In the context of a population based survey on attitudes of the general German population towards medical second opinions we conducted a SWAT using a parallel group, randomized controlled trial with a 1:1 allocation. For details please consult the original paper [15]. The survey is part of a mixed method study on medical second opinions in Germany [16]. We describe the design of the original study in brief: We randomly identified 9990 individuals via 25 randomly selected population registration offices in the states of Berlin and Brandenburg. We selected these two states because they cover a settlement structure with both very high but also very low population density. Participants were selected through a disproportionate stratified sample, with settlement structure (cities, towns and suburbs, rural areas) serving as the stratification variable, resulting in three equally sized samples. Settlement patterns are defined by “degree of urbanization” of Eurostat [17]. The register offices belonged to cities (*n* = 5), towns and suburbs (*n* = 10) and rural areas (*n* = 10). There are only 5 urban areas in those federal states, which is why we chose all of them. See Fig. 1 for the detailed process. Moreover the register offices contained data on gender and most on age as well. The questionnaire contained 47 items on 14 pages. It included 8 parts: health-related items (2 items), local medical care situation (5 items), patients’ needs concerning second opinions (7 items), potential experiences with second opinions (8 items), design of the second opinion procedure (8 items), experiences with and knowledge of second opinion programs by health insurers (3 items), experiences with and knowledge of the offer of second opinion provider (3 items). The last part included sociodemographic characteristics (11 items). We used the European Health Literacy Survey including 16 items to assess health literacy [18].

### Participants

As this was a population based survey we had no eligibility criteria except age (≥18 years) and residence in either Berlin or Brandenburg. The questionnaire was send via post twice (initially and 6 weeks later with a reminder) together with the invitation to participate. The questionnaire could be send back to us with business-reply envelopes. By returning the questionnaire to us, participants could also include their declaration of consent to take part in a lottery to win one of 125 Amazon vouchers (50€ each) in a separate envelope.

### Interventions

The intervention group received an invitation letter with a colored photograph of two female researchers, and the control group received an invitation letter without a photograph. Otherwise the letters were identical. Please see Additional file 1 for details.

Originally, we planned to perform a factorial design with two different interventions in a 1:1:1:1 manner. The second intervention was a teaser on the envelope (“Join now and help shape healthcare”) compared to an unprinted envelope. Unfortunately, the printing company only sent unprinted envelopes, so that we did not follow up on this.

### Outcomes

The outcome was the participation rate. The participation rate is defined as the number of people who answered at least one question relative to the total number of those who received an invitation to the survey. Whereas response rate is defined as the number of people who respond to the survey in any way, relative to the total number of recipients of the survey invitation. The response rate can always be higher than the participation rate a refusal to participate would be a response but not a participant.

### Sample size

All of the addressees of our survey on second medical opinions were included in this SWAT. We chose an equally sized disproportionate stratified sample (cities, towns and suburbs, rural area) as we hypothesized that there might be differences regarding our initial study question (second opinions) between the settlement patterns.

### Randomization

For randomization, one researcher used random numbers generated by Microsoft Excel. Participants were originally allocated in a 1:1:1:1 manner without stratification. In group 1: cover letter without photograph and without a teaser on the envelope, group 2: cover letter with photograph without a teaser on the envelope, group 3: cover letter without photograph with a teaser on the envelope, group 4: cover letter with photograph with a teaser on the envelope. The same researcher who determined the allocation sent the address data of the four groups to the printing company. As soon as we found out that the printing of the envelopes with a teaser had failed, we merged group 1 and group 3 to group 1 and group 2 and group 4 to group 2.

### Blinding

The participants were not aware that we conducted this SWAT within our survey. There was only a small mark on the bottom of the questionnaire “I”, “II”, “III”, “*blank space* “for the groups 1, 2, 3 and 4 so that researchers could identify in which group the participants were allocated. The researchers were not blinded for data analysis.

### Data acquisition

All data received in the survey were extracted in an excel spreadsheet. And data form the whole sample, received from the registration offices (gender, postal code, and age for most of the population registration offices), were added to a second excel spreadsheet. We categorized postal codes according to the ‘degree of urbanisation’ of Eurostat and regional statistics by the German Federal Statistical Office, which are in accordance with the settlement patterns described earlier [17, 19]. To create international comparability we used the Comparative Analysis of Social Mobility in Industrial Nations (CASMIN) categorization to abstract education. CASMIN contains three main categories, primary, secondary and tertiary education with various subcategories. We used the categories tertiary education (academic degree, independent of school education and type of degree), higher secondary (no or any vocational education with at least intermediate school education) and lower secondary (no or any vocational education with at least general school education) [20]. Due to the study design, we were not able to collect the reasons for non-participation systematically.

### Statistical methods

We calculated the risk difference and the odds ratio for the primary outcome using contingency tables. Differences between characteristics of participants in both groups were tested by performing *χ*^2^ analyses for binary data and t-test for continuous data. We performed exploratory subgroup analyses in women, men, those who lived in cities, in towns and suburbs and in rural areas, comparing participants and non-participants.

In the subgroup analyses we also used *χ*^2^ analyses for binary data. We planned to use logistic regressions but were not able to do so, as individual data of participants was not fully assignable. All analyses were performed in SPSS version 28.0 and Microsoft Excel.

## Results

### Participant flow

On the 6th of March 2020 we sent 9990 invitations and the reminders on the 24th of April. Of the initial 9990 participants 193 could still not be reached after 2 attempts, reasons were for example “receiver unknown” or “moved without possibility to forward to new address”. We assumed that the questionnaire could be delivered to N 9797 persons, since it was not returned. However, we could not be sure if it was successfully received. See Table 1 for baseline characteristics.

**Table 1:** Baseline Characteristics of those where invitation was not returned

	With photograph n (%) *N* = 4890	Without photograph n (%) *N* = 4907
*Females*	2506 (51.3)	2444 (49.8)
*Cities*	1612 (33.0)	1612 (32.9)
*Towns and suburbs*	1633 (33.4)	1650 (33.6)
*Rural areas*	1645 (33.6)	1645 (33.5)
	**N, mean (SD)**	**N, mean (SD)**
*Age in years*	4313^a^	4345^a^
	55.3 (18.5)	55.1 (18.3)

^a^Participants from 3 population registration offices needed to be removed from this analysis as age was not transmitted from these

Of these 9797, 1349 persons participated in our survey (participation rate of 13.8%, 1349/9797), see flow chart in Fig. 1. We received the last envelope in July 2020. In the group “with photograph” 682 (13.9%, 682/4890) people participated and in the group “without photograph” 667 (13.6%, 667/4907) people participated resulting in an absolute risk difference of − 0.36% (95% CI -1.719-1.011) and an odds ratio (OR) of 1.030 (95% CI 0.918–1.156). There was no relevant difference between the intervention group and the control group in any of the queried characteristics. See Table 2 for details on characteristics of participants.

**Table 2:** Characteristics of participants

	With photograph n (%) *N* = 4890	Without photograph n (%) *N* = 4907	***P***-value
*Participation rate*^a^	682 (13.9)	667 (13.6)	0.611
*Gender*	**678**	**660**	
Females	390 (57.5)	368 (55.6)	0.515
*Housing status*	**668**	**644**	0.950
Living alone	168 (25.1)	161 (25)	
Living with at least one more person	500 (74.9)	483 (75.0)	
*Education level according to CASMIN*	**650**	**630**	0.234
Lower secondary education	57 (8.8)	73 (11.6)	
Higher secondary education	352 (54.2)	325 (51.6)	
Tertiary education	241 (37.1)	232 (36.8)	
*Settlement pattern*	**661**	**646**	0.582
Cities	258 (39.0)	253 (39.2)	
Rural areas	217 (32.8)	226 (35.0)	
Towns and suburbs	186 (28.1)	167 (25.9)	
	**N, mean (SD)**	**N, mean (SD)**	
*Age in years*	**665**	**644**	
	56.0 (17.0)	57.2 (16.4)	0.353

*CASMIN* Comparative Analysis of Social Mobility in Industrial Nations

^a^Primary outcome

Within a subgroup analyses of women, men, those who lived in cities, in towns and suburbs and in rural areas, we compared participants and non-participants. We saw neither in women nor in men a difference in participation. Furthermore, the participation rate was similar regarding settlement pattern. Even though we measured a little higher participation rate in the group with photograph in people living in towns and cities and a little lower participation rate in people living in rural areas, there was no relevant difference, see Table 3 for further details.

**Table 3:** Results of the subgroup analyses

	With photograph n (%)	Without photograph n (%)	***P***-value
*Females*	2506	2444	0.622
Participants	390 (15.6)	368 (15.1)	
Non-Participants	2116 (84.4)	2076 (84.9)	
*Males*	2383	2463	0.190
Participants	288 (12.1)	292 (11.9)	
Non-Participants	2096 (87.9)	2171 (88.1)	
*Cities*	1612	1612	0.809
Participants	258 (16.0)	253 (15.7)	
Non-Participants	1354 (84.0)	1359 (84.3)	
*Towns and suburbs*	1633	1650	0.241
Participants	186 (11.4)	167 (10.1)	
Non-Participants	1447 (88.6)	1483 (89.9)	
*Rural areas*	1645	1645	0.646
Participants	217 (13.2)	226 (13.7)	
Non-Participants	1428 (86.8)	1419 (86.3)	

Participants from 3 population registration offices needed to be removed from this analysis as age was not transmitted from these

## Discussion

We conducted a SWAT to compare the effectiveness of invitation letters *with* a photograph of the researcher with that of invitation letters *without* a photograph of the researcher, as part of a survey of attitudes towards medical second opinions. We found no difference in the participation rate due to the use of a photograph. We neither found any differences when we compared gender and settlement patterns of participants and non-participants. We also planned to examine another intervention, a teaser “Join now and help shape healthcare” printed on the envelope. Unfortunately, all envelopes were sent out without this teaser by the printing company. Overall, the implementation of the SWAT caused no additional costs and very little additional work for the preparation of the invitation letter and the teaser.

In two other studies which used a photograph of the researcher on the invitation letter, results were contradictory. While one study reported a participation rate of29.3% and an OR of 2.9 (95% CI 1.5–6.1) for the photograph group (40.0% participation versus 19.0% participation), the other study reported in total 54.0% participation rate with 30.0% in both groups before a reminder and 50.0% in the group with photograph and 63.0% without photograph after a reminder [21, 22]. Both studies differed to ours in various aspects. Dommeyer et al. from 1997 surveyed 150 persons on music censorship using a 4-page questionnaire. The invitation letter was only half a page and was written from the perspective of a college student. 44 other college students rated the photo for physical attractiveness and sex appeal before the survey [21]. In 1984, Rucker et al. asked 349 graduate students from the same university to answer a questionnaire on furniture. They used either a picture of the researcher in a formal dress, in casual clothes or no picture at all [22]. Not only the setting of both studies and content of the surveys differed but also the methods used. Therefore, the studies are difficult to compare amongst each other as well as with our work. However, there are various reasons possible why results differed that much; one is the rating of attractiveness of the picture utilized by Dommeyer et al.. Gueguen et al. showed that the use of an attractive photograph increases the respone rate compared to a medium attractive photograph in an email survey [23]. In Rucker et al. the personalization is generally higher, since graduates from the same university were contacted and all addresses were handwritten on the envelopes, which is expected to increase the participation rate [12]. The authors assumed that there is a point of “overpersonalization”. They state that subjects reacted negatively (with non-participation) to the higher personalized conditions, especially after the reminder was sent, and assume it was “too much” [22]. Another possible explanation is that the attitude towards personalization through a photograph has transformed over the last 30 years, as communication changed a lot and the use of photographs is nowadays familiar. Moreover, our sample was much larger than that of both other studies. This might be due to the fact that their samples were too small to show the true effect. According to the Leverage Salient Theory, individuals assign varying importance to different aspects of a survey. Some are attracted by the topic, some by the sender and some by the incentive [24]. If researchers put an emphasis on one of these aspects, they may influence who will participate to a small extent. The emphasis cannot be generalized because the value of each aspect varies in a population [25]. Given our results and the findings from the Cochrane review by Edwards et al., the question of individualized recruitment strategies rather than a one fits all solution is pressing. Future research should consider under what conditions and for whom a particular aspect of the survey has an impact with the aim to use this information in recruiting strategies [25]. We did not aim to emphasize any particular aspect in our survey. As stated above, a non-monetary strategy is inexpensive and easy to implement. When conducting a study or survey one should always consider if a SWAT is possible and useful.

### Limitations

Our SWAT has several limitations. We assume that the low participation rate of 13.8% could be a factor that leads to the possible effects of a recruitment measure being overshadowed. One possible reason for the low participation rate is the length of our survey. Filling out our questionnaire took about 30 to 40 minutes. In a survey of physicians, Jepson et al. showed a decrease from a 60% response rate for questionnaires with 849 words to 16.7% for questionnaires with more than 1800 words [26]. The participation rate tends to be higher when one is affected by a topic him- or herself or in general with a “more interesting” topic [12]. Our target group was the general population and it was not clear how many people already had experience or interest in medical second opinions. However, it is a topic which may affect anyone. Furthermore, we used a mixture of attitudinal and factual questions. Cartwright et al. showed that factual questions lead to higher participation rates than mixed questions [27]. As an incentive, we used a lottery. Those who participated had the chance to win a 50 Euro Amazon voucher (we raffled 125 vouchers). Interestingly, only 70% of the participants took part in the lottery. We expected a higher rate, but some people criticized the use of Amazon vouchers as they had objections against Amazon as a company. In principle, the use of prepaid cash incentives has shown to be the most useful means to increase participation rates [28]. As our funding was limited, wewere not able to give a prepaid incentive to everyone. In addition, Gajic et al. found that although participation rates were higher for a prepaid incentive, the higher the lottery prize, the more participants returned completed questionnaires [29]. Unfortunately, we were not able to check our second intervention, the teaser, because the printing company sent all the envelopes unprinted by mistake. Our expectation were higher participation rates through the use of the teaser [30]. The first round of invitation letters was sent during the first weeks of a COVID-19 induced lockdown. It is unclear whether and how this affected our results. Furthermore, the timeframe between the first invitation and the reminder was 7 weeks. One reason for that long period between first contact and reminder was that we had about 400 invitations sent back to us because of an error in the addressees, which we sent out again. We wanted to be sure that there was an interval of at least 4 weeks between the first invitation and the reminder for all recipients. We have not found any evidence on what is the best period to send one or more reminders, but at least a recommendation to send the first reminder after three weeks and to send more than one reminder [31, 32].

### Future directions

In general, we were surprised that we did not find any other SWAT or other recent studies on the same topic, as recruitment is a highly relevant topic and performing a SWAT like we did is quite cost effective. We imply, considering our results, that there might be other SWATs on the topic resulting in no effect which were not published.

## Conclusion

We did not find any difference in the participation rate when we used a photograph of the researcher in the invitation letter or chose not to do so. We neither saw any difference of sociodemographic factors in the non-participant analysis. More research on recruitment for surveys with low funding is needed. It is easy and inexpensive to add a photo of the researchers to the invitation letter when conducting a survey. Although we did not find an increase in participation rate, we also did not find any negative effects of using a photograph.

## Supplementary Information

**Additional file 1.**

## Data Availability

The datasets generated and analysed during the current study will be available from the corresponding author on reasonable request.

## Abbreviations

CASMIN: Comparative Analysis of Social Mobility in Industrial Nations
CG: Control group
IG: Intervention group
OR: Odds ratio
SWAT: Study within a trial
