# Supplementary material for: A photograph of the researcher on the invitation letter did not affect the participation rate of a postal survey: a randomized study within a trial (SWAT)
Source: BMC Med Res Methodol. 2022 Sep 24;22:249. doi: 10.1186/s12874-022-01717-3 (PMC9508740; doi:10.1186/s12874-022-01717-3)
Supplement: Supplementary file 1 — Additional file 1. [file 12874_2022_1717_MOESM1_ESM.pdf]

Universität Witten/Herdecke
IFOM • Ostmerheimer Straße 200 • 51109 Köln

06.03.2020

Ihre Gesundheit ist Ihnen wichtig? Gemeinsam mit Ihrer Erfahrung können wir unser Gesundheitssystem verbessern.

Sehr geehrte/r Frau X/Herr X,

wir freuen uns, Sie über unsere Studie zum Thema zweite ärztliche Meinung in Deutschland zu informieren, und laden Sie herzlich ein, daran teilzunehmen!

Wie viele Angebote holen Sie sich bei einem Autokauf oder der nächsten Urlaubsreise ein? Vertrauen Sie bei einem Rechtsstreit nur auf Ihre eigene Meinung oder holen Sie noch einen rechtlichen Beistand hinzu? Gehen Sie mit Ihrer Gesundheit genauso vorsichtig um?

<u>Worum es in unserer Studie geht:</u>

- Haben Sie schon einmal eine Diagnose oder Behandlungsempfehlung von Ihrem Arzt erhalten, die Sie von einem zweiten Arzt abklären lassen wollten? Vielleicht hatten Sie aber auch noch nie das Bedürfnis, eine zweite ärztliche Meinung einzuholen?
- Wir, die **Universität Witten/Herdecke (UW/H),** benötigen **Meinungen von Bürgern,** um Vorteile, Nachteile und Wünsche zu zweiten ärztlichen Meinungen herauszufinden – **dazu müssen Sie aber keine zweite ärztliche Meinung eingeholt haben.**
- Unsere Studie wird öffentlich gefördert. Weitere Informationen zu unserem Projekt **ZWEIT** finden Sie unter: https://innovationsfonds.g-ba.de/

<u>Wie Sie teilnehmen können:</u>

- Sie wurden **zufällig aus den Melderegister-Daten des Landes Berlin oder Brandenburg** ausgewählt.
- Anbei befindet sich ein Fragebogen. Das Ausfüllen dauert ca. 20 - 30 Minuten.
- Unter allen Teilnehmern werden **125 Amazon Gutscheine zu je 50 €** verlost.
- Füllen Sie für die Teilnahme am Gewinnspiel die Einwilligungserklärung aus (letzte Seite

des Fragebogens) und schicken Sie uns diese zusammen mit dem ausgefüllten Fragebogen in dem beigefügten **adressierten und frankierten Rücksendeumschlag** zurück.
- Damit leisten Sie einen **wichtigen Beitrag zur Verbesserung der Gesundheitsversorgung**!

<u>Wie geht es weiter:</u>
- Die Befragung erfolgt anonym. Für die Teilnahme am Gewinnspiel ist es nötig, dass Sie Ihren Namen und Ihre Adresse auf der Einwilligungserklärung (letzte Seite) angeben. Diese wird gesondert von den Fragebögen aufbewahrt. Die Teilnahme ist selbstverständlich freiwillig und eine Nicht-Teilnahme wirkt sich für Sie in keiner Weise negativ aus.
- Für weitere Informationen bezüglich des Datenschutzes ziehen Sie bitte die Einwilligungserklärung auf der letzten Seite zu Rate.
- Wir werden Sie ein weiteres Mal postalisch kontaktieren, um sicherzugehen, dass unser Anschreiben Sie erreicht hat.

Wir würden uns sehr freuen, wenn Sie unsere Studie zur Weiterentwicklung von zweiten ärztlichen Meinungen unterstützen. Vielen herzlichen Dank!

Nadja Könsgen
Wissenschaftliche Mitarbeiterin UW/H
Tel.: +49 (0)221 / 98957-50
E-Mail: nadja.koensgen@uni-wh.de

Barbara Prediger
Wissenschaftliche Mitarbeiterin UW/H
Tel.: +49 (0)221 / 98957-44
E-Mail: barbara.prediger@uni-wh.de

Mit freundlichen Grüßen

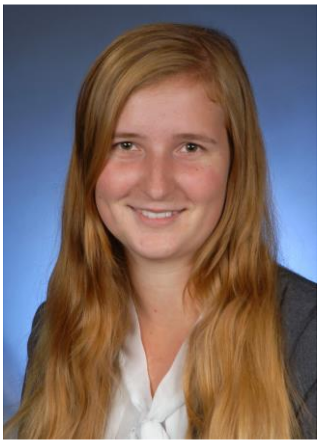

Nadja Könsgen

Nadja Könsgen

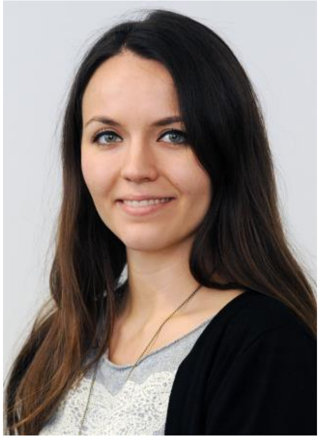

B. Pred

Barbara Prediger

Sie benötigen weitere Informationen zu unserer Studie? Einfach QR-Code scannen:

Universität Witten/Herdecke
IFOM • Ostmerheimer Straße 200 • 51109 Köln

06.03.2020

Ihre Gesundheit ist Ihnen wichtig? Gemeinsam mit Ihrer Erfahrung können wir unser Gesundheitssystem verbessern.

Sehr geehrte/r Frau X/Herr X,

wir freuen uns, Sie über unsere Studie zum Thema zweite ärztliche Meinung in Deutschland zu informieren, und laden Sie herzlich ein, daran teilzunehmen!

Wie viele Angebote holen Sie sich bei einem Autokauf oder der nächsten Urlaubsreise ein? Vertrauen Sie bei einem Rechtsstreit nur auf Ihre eigene Meinung oder holen Sie noch einen rechtlichen Beistand hinzu? Gehen Sie mit Ihrer Gesundheit genauso vorsichtig um?

<u>Worum es in unserer Studie geht:</u>

- Haben Sie schon einmal eine Diagnose oder Behandlungsempfehlung von Ihrem Arzt erhalten, die Sie von einem zweiten Arzt abklären lassen wollten? Vielleicht hatten Sie aber auch noch nie das Bedürfnis, eine zweite ärztliche Meinung einzuholen?
- Wir, die **Universität Witten/Herdecke (UW/H),** benötigen **Meinungen von Bürgern,** um Vorteile, Nachteile und Wünsche zu zweiten ärztlichen Meinungen herauszufinden – **dazu müssen Sie aber keine zweite ärztliche Meinung eingeholt haben.**
- Unsere Studie wird öffentlich gefördert. Weitere Informationen zu unserem Projekt **ZWEIT** finden Sie unter: https://innovationsfonds.g-ba.de/

<u>Wie Sie teilnehmen können:</u>

- Sie wurden **zufällig aus den Melderegister-Daten des Landes Berlin oder Brandenburg** ausgewählt.
- Anbei befindet sich ein Fragebogen. Das Ausfüllen dauert ca. 20 - 30 Minuten.
- Unter allen Teilnehmern werden **125 Amazon Gutscheine zu je 50 €** verlost.
- Füllen Sie für die Teilnahme am Gewinnspiel die Einwilligungserklärung aus (letzte Seite

des Fragebogens) und schicken Sie uns diese zusammen mit dem ausgefüllten Fragebogen in dem beigefügten **adressierten und frankierten Rücksendeumschlag** zurück.

- Damit leisten Sie einen **wichtigen Beitrag zur Verbesserung der Gesundheitsversorgung**!

<u>Wie geht es weiter:</u>

- Die Befragung erfolgt anonym. Für die Teilnahme am Gewinnspiel ist es nötig, dass Sie Ihren Namen und Ihre Adresse auf der Einwilligungserklärung (letzte Seite) angeben. Diese wird gesondert von den Fragebögen aufbewahrt. Die Teilnahme ist selbstverständlich freiwillig und eine Nicht-Teilnahme wirkt sich für Sie in keiner Weise negativ aus.
- Für weitere Informationen bezüglich des Datenschutzes ziehen Sie bitte die Einwilligungserklärung auf der letzten Seite zu Rate.
- Wir werden Sie ein weiteres Mal postalisch kontaktieren, um sicherzugehen, dass unser Anschreiben Sie erreicht hat.

Wir würden uns sehr freuen, wenn Sie unsere Studie zur Weiterentwicklung von zweiten ärztlichen Meinungen unterstützen. Vielen herzlichen Dank!

Nadja Könsgen
Wissenschaftliche Mitarbeiterin UW/H
Tel.: +49 (0)221 / 98957-50
E-Mail: nadja.koensgen@uni-wh.de

Barbara Prediger
Wissenschaftliche Mitarbeiterin UW/H
Tel.: +49 (0)221 / 98957-44
E-Mail: barbara.prediger@uni-wh.de

Mit freundlichen Grüßen

Nadja Könsgen

Barbara Prediger

Sie benötigen weitere Informationen zu unserer Studie? Einfach QR-Code scannen:
